# Ethical issues in the treatment of extremely low birth weight neonates

**DOI:** 10.3325/cmj.2016.57.395

**Published:** 2016-08

**Authors:** Aleksandra Doronjski, Vesna Stojanović

**Affiliations:** Institute for Child and Youth Health Care of Vojvodina, Medical Faculty, University of Novi Sad, Novi Sad, Serbia

Doronjski and Stojanović: Ethical issues in the treatment of extremely low birth weight neonates

During their everyday work in neonatal intensive care unit (NICU), physicians face many ethical dilemmas. Modern technology increases the chances of survival even of extremely low birth weight (ELBW) newborns, with a minimum number of complications. In countries with guides and expert group recommendations, neonatologists’ decisions on limiting or withdrawing life-sustaining medical treatment (LSMT) in critically ill patients are led by these recommendations. However, what should be done in a country where such recommendations are not available?

In Serbia there are no written recommendations, either in the form of government legislation or in the form of perinatal/neonatal association guidelines. The only suggestion made by an informal group of neonatologists is to resuscitate neonates born after 23 gestational weeks (GW) and those weighting more than 400 g at birth.

## How is this issue dealt with in other countries?

The mortality of ELBW neonates is still high in some parts of the world. According to the American College of Obstetrics and Gynecology data, in neonates born before 21 GW no survival has been reported, in neonates born before 24 GW survival is low and is followed by severe neurological damage, while in neonates born at 25 GW the survival rate is up to 75% (1)!

The basic ethical dilemma is who should be the one to decide whether an ELBW neonate should be resuscitated after birth and if it survives whether it will be treated according to all protocols of good clinical practice, or it will be given only palliative care. Most physicians think that it is them rather than the parents who should make the main decisions. On the other hand, parents think they should be the ones with the main role in decision making. This is why “Negotiated” model is used in the best interest of the patient (2).

A study in neonatal intensive care units in Scotland found that 56% of parents thought they should participate in decision making concerning LSMT limitation or withdrawal, and two thirds thought they should make the decision themselves regardless of the physician’s opinion (3). In an international study group (nine centers from Pacific Rim countries and two centers from San Francisco), 93-100% parents of the children who survived with very low birth weight respected the physician’s decisions concerning the need for resuscitation. The majority of the parents (65-90%) considered that the decision on further treatment should be carried out by the parents and physicians together. In Melbourne, three quarters of parents considered that the physician should be the only one to make the decision (4), while in Norway, most of the parents thought that physicians’ opinions were more important concerning the “end of life” decisions, but that the parents too should be included (5). In a Canadian study, almost all parents thought that they should have the last word about starting and/or limiting the treatment (6).

Ethically, physicians have a duty to inform the newborn’s parents about resuscitation procedures and the potential outcome and to ask for consent for treatment and other procedures. In most of the developed countries antenatal consultation is provided, where all the possible aspects of newborn treatment, all the benefits and risks of resuscitation, and all possible outcomes and consequences are explained to the parent before the birth of an ELBW neonate. The moral obligation of the physician is to act in the best interest of the patient. When it is difficult to make a decision, parents’ wishes must be considered (7). It would be best to make a decision about further therapeutic procedures right after birth.

The laws of some states (Wisconsin, USA) state that withholding or withdrawing LSMT is not in the best interest of the patient unless the patient is in a persistent vegetative state. In the absence of a persistent vegetative state, parents do not have the right to withhold or withdraw LSMT of their critically ill newborn (8).

In the USA and Western countries, the stances on this question are the following: resuscitate newborns born after 24 GW and do not resuscitate newborns born before 23 GW. For newborns born between 23 and 24 GW, parents’ wishes are taken in consideration, and outside this range, parents’ wishes have little influence on further treatment. The most important factors that affect the decision whether the reanimation will be used are GW, parents’ wishes, neonate’s birth weight, and the presence of anomalies (9).

In the USA and most of Western countries, LSMT is withheld or withdrawn in newborns who are moribund and estimated to die regardless of the therapeutic measures used, but also in the cases when the long-term quality of life will evidently be bad. In these cases, therapy of pain and palliative care are used. The length of life of non-survivor ELBW neonates extended from 2 days in 1991 to 10 days in 2001 (10).

## The current situation in Serbia

In Novi Sad, maternity clinic is physically separated from our NICU (where newborns in critical condition are transported), hence the small number of neonatologists working there do not provide antenatal consultation. Accordingly, after the birth of an ELBW neonate, the physician is the only one who decides about the usage of resuscitation and further treatment of the newborn. Also, the fathers are not allowed into the maternity hospital, and the mother is often unable to make such complex decisions during or right after birth.

During neonatal transfer from the maternity ward to the level III NICU, the pediatrician gives the baby’s mother only the necessary information about the hospital to which the baby is being transported. However, there is not enough time to discuss the parents’ expectations and wishes. There is also no time for the decision making topic at all. The synchronization between the neonatologist at the maternity ward and the pediatrician in level III NICU is very poor. We believe this is the key reason why the parents are absent from the decision making process. If the parents were informed about the fetal/neonatal condition and prognosis prior or immediately after birth, there would probably be enough time for them to form an opinion on their child’s future treatment. In the absence of the collaboration between the maternity ward physicians and level III NICU physicians, the parents are reduced to mere spectators.

Based on the existing recommendations on the minimal birth weight and genetic age of newborns, the neonatologists in the Maternity Hospital have recently decided to resuscitate and treat two ELBW neonates. The first neonate had BW over 400 g and was born at 22 GW and the second had very low BW (330 g), but was born at 25 GW. When our transport crew arrived both neonates had stable vital parameters and were on mechanical ventilation. They were transported to our NICU and the treatment was continued, including surfactant administration, mechanical ventilation, antibiotic therapy, and other supportive and substitution therapies. Ethical dilemmas occurred a few days later, when both patients’ general condition worsened. Parents did not contact us for consultation or information. We decided to limit the LSMT of the first newborn since his general condition was severe and he had very low gestational age. The second patient was diagnosed with acute kidney injury on the 4th day of life, and after having performed all the therapeutic measurements, peritoneal dialysis was started. Were we unrealistic since the patient was born at 25 GW? Would we have done something different if the parents had been available and included into decision making? Since there are no clear indications for treating or ceasing to treat ELBW newborns in our country, the physician is the only one who makes the decision about limiting or withdrawing LSMT.

On the other hand, it is very hard to make a decision about terminating a treatment conducted in accordance with all protocols. In the Netherlands, a country with liberal regulation concerning active life termination, 70% of all neonatal deaths are caused by life termination decisions. Despite this, this percentage has not increased in decades, which proves that physicians have a very hard time deciding when to terminate a treatment (11).

It is very difficult to predict the outcome for the most of ELBW neonates, which additionally impedes the decision on starting the treatment. If the mother did not visit her gynecologist regularly, it is hard to estimate the real gestational age. On the other hand, BW depends on many factors and is often not a reflection of maturity, and many newborns with the same BW often have different survival chances. Even in the case when this information is available and precise, it is still hard to decide because some patients with very low body weight and gestational age have survived with very few complications. This leads to the question what risk is acceptable and who should decide about it.

The general stance on this is that the decisions in medical interventions for the neonates born between 23 and 24 +6/7 GW should be made based on patient’s clinical state, response to therapy, parents’ wishes, and neonatologist’s opinion. The biggest moral issue during the decision-making about further treatment is its influence on the family of the child who might have severe neurological consequences (12,13).

These ethical dilemmas and questions should not be regulated by law. Perinatology societies in many countries have issued recommendations regulating the minimal body weight and gestational age of a newborn, in order to have resuscitation performed after birth (14-17). In most countries there are no such recommendations. In our country, societies do not have such legitimacy, and parents have no influence on decision making. The decision about treating or not treating ELBW neonates should not be conditioned by the physician’s fear of punishment, but should be made in the patient’s best interest. No decision made in this way will be against the law.

In order to overcome these dilemmas, the parents and physicians should be equally involved in decision making process about limiting or withdrawing LSMT. This is only doable if expert associations issue clear recommendations based on good medical practice and have them backed up by law. Every parent should be informed in detail about the ELBW newborns’ treatment results, and in order to do that, there must be a good cooperation between gynecologist-obstetricians and the level III NICU neonatologists.

## References

[1] American College of Obstetrics and Gynecology (2002). Perinatal care at the threshold of viability. ACOG Practice Bulletin.

[2] McHaffie HE, Laing IA, Parker M, McMillan J (2001). Deciding for imperilled newborns: medical authority or parental autonomy?. J Med Ethics.

[3] McHaffie HE, Lyon AJ, Hume R (2001). Deciding on treatment limitation for neonates: the parents’ perspective.. Eur J Pediatr.

[4] Partridge JC, Martinez AM, Nishida H, Boo NY, Tan KW, Yeung CY (2005). International comparison of care for very low birth weight infants: parents’ perceptions of counseling and decision-making.. Pediatrics.

[5] Brinchmann BS (2002). What matters to the parents? A qualitative study of parents’ experiences with life-and-death decisions concerning their premature infants.. Nurs Ethics.

[6] Streiner DL, Saigal S, Burrows E, Stoskopf B, Rosenbaum P (2001). Attitudes of parents and health care professionals toward active treatment of extremely premature infants.. Pediatrics.

[7] Leuthner SR (2001). Decisions regarding resuscitation of the extremely premature infant and models of best interest.. J Perinatol.

[8] Montalvo v. Borkovec. In: WI App 147:256 Wis. 2d 472; 2002.

[9] De Leeuw R, Cuttini M, Nadai M (2000). Treatment choices for extremely preterm infants: an international perspective.. J Pediatr.

[10] Meadow W, Lee G, Lin K, Lantos J (2004). Changes in mortality for extremely low birth weight infants in the 1990s: implications for treatment decisions and resource use.. Pediatrics.

[11] Vrakking AM, van der Heide A, Onwuteaka-Philipsen BD, Keij-Deerenberg IM, van der Maas PJ, van der Wal G (2005). Medical end-of-life decisions made for neonates and infants in the Netherlands, 1995–2001.. Lancet.

[12] Jones PM, Carter SB (2008). Resuscitating the extremely low-birth-weight infant: Humanitarianism or hubris?. Virtual Mentor.

[13] Ambalavanan N, Carlo WA, Bobashev G (2005). Prediction of death for extremely low birth weight neonates.. Pediatrics.

[14] Nadroo AM (2011). Ethical dilemmas in decision making at limits of neonatal viability.. JIMA.

[15] Seri I, Evans J (2008). Limits of viability:definition of the gray zone.. J Perinatol.

[16] Kelley M, Rubens CE (2010). GAPPS review group. Global report on preterm birth and stillbirth (6 of 7):ethical considerations.. BMC Pregnancy Childbirth.

[17] Ho LY, Tan TK, Goh JPS (2013). Advocating for the best interest of critically ill newborns infants. Proceedings of Singapore Healthcare.

